# Causal associations between gut microbiota and five common oral lesions: A two-sample Mendelian randomization study

**DOI:** 10.1097/MD.0000000000049705

**Published:** 2026-08-07

**Authors:** Ying Tang, Jianming Yuan, Feng Pan

**Affiliations:** a Department of Prosthodontics, Huangpu District Dental Disease Prevention and Treatment Institute, Shanghai, China.

**Keywords:** Causal associations, genome-wide association studies, gut microbiota, mendelian randomization, oral lesions

## Abstract

Previous studies have investigated the relationship between gut microbiota (GM) and oral lesions, but the reported associations across microbial taxa and oral conditions have been inconsistent. This 2-sample Mendelian randomization study using summary-level GWAS data aimed to clarify the causal links between GM and 5 oral conditions: loose teeth, toothache, bleeding gums, painful gums, and mouth ulcers. GM data were obtained from the MiBioGen consortium, while outcome data were derived from the Neale Lab and MRC-IEU consortium. We applied inverse variance weighted (IVW) and complementary MR methods to estimate causal effects, with reverse MR performed to test the possibility of reverse causality, and multivariable MR (MVMR) used to assess whether associations persisted after adjusting for conventional risk factors. The IVW method identified 42 potential associations between GM and oral conditions. For example, the Mollicutes class and Tenericutes phylum were negatively associated with loose teeth (Mollicutes: odds ratio [OR] = 0.9936, 95% confidence interval [CI]: 0.9894 to 0.9978, *P* = .0029), whereas the Slackia genus showed a positive association with toothache (OR = 1.0096, 95% CI: 1.0050 to 1.0142, *P* = 3.98 × 10^−5^). These associations were further confirmed in replication analyses, and MVMR demonstrated that they remained significant after accounting for established risk factors. The findings suggest robust causal links between specific GM taxa and oral conditions, offering novel insights into the GM–oral health axis and highlighting potential preventive or therapeutic targets for oral care.

## 1. Introduction

The oral cavity, as the initial segment of the digestive system, plays a critical role in digestion, mastication, swallowing, speech, and sensory perception.^[1]^ Despite its importance, oral health has long been underappreciated, and the burden of oral lesions is considerable, particularly in aging populations.^[2]^ Epidemiological data show that 12% of American children aged 6 to 12 years experience toothache,^[3]^ and mouth ulcers affect 10–20% of the population at some point in life, with higher prevalence among older adults.^[4,5]^ These lesions are associated with pain, impaired quality of life, and psychological distress, while current treatments largely focus on symptomatic relief without preventing recurrence or progression.^[6–10]^ Therefore, identifying causal mechanisms underlying oral lesions is essential for effective prevention and intervention.

The gut microbiota (GM), composed of trillions of microorganisms, performs vital functions in nutrient metabolism, immune regulation, and epithelial barrier maintenance.^[11–13]^ Growing evidence supports the concept of a gut–oral axis, whereby alterations in GM influence oral health through systemic inflammatory and metabolic pathways. Beyond periodontitis, recent studies suggest that GM shifts may contribute to prosthodontic complications, oral mucositis, oral cancer and recurrent ulceration.^[14–18]^ For instance, translocation of pathogenic taxa such as Porphyromonas gingivalis has been implicated in both periodontal and non-periodontal oral lesions, while probiotic interventions targeting GM have shown benefits in alleviating mucosal inflammation.^[19–21]^ Nevertheless, these studies are primarily observational, leaving unresolved whether GM alterations are causal drivers of oral lesions or secondary to disease progression.

To overcome the limitations of observational studies, including reverse causality and residual confounding, Mendelian randomization (MR) provides a powerful genetic epidemiological approach to assess causality. By using genetic variants as instrumental variables for GM traits, MR can mitigate biases and enable stronger inference.^[22–24]^ Previous MR studies have investigated risk factors for periodontitis and other oral conditions,^[25,26]^ supporting its utility in oral epidemiology. In this study, we focused on 5 representative oral lesions, loose teeth, toothache, bleeding gums, painful gums, and mouth ulcers. These were selected because they are common, clinically impactful, and capture a spectrum of inflammatory and degenerative oral pathologies. In contrast, other oral conditions such as xerostomia and candidiasis were excluded due to limited GWAS data availability and different etiological pathways. We hypothesized that genetically predicted alterations in GM composition are causally associated with these oral lesions, and that MR would provide robust genetic evidence to support or refute these associations.

## 2. Methods

### 2.1. Study design

**Figure 1.**
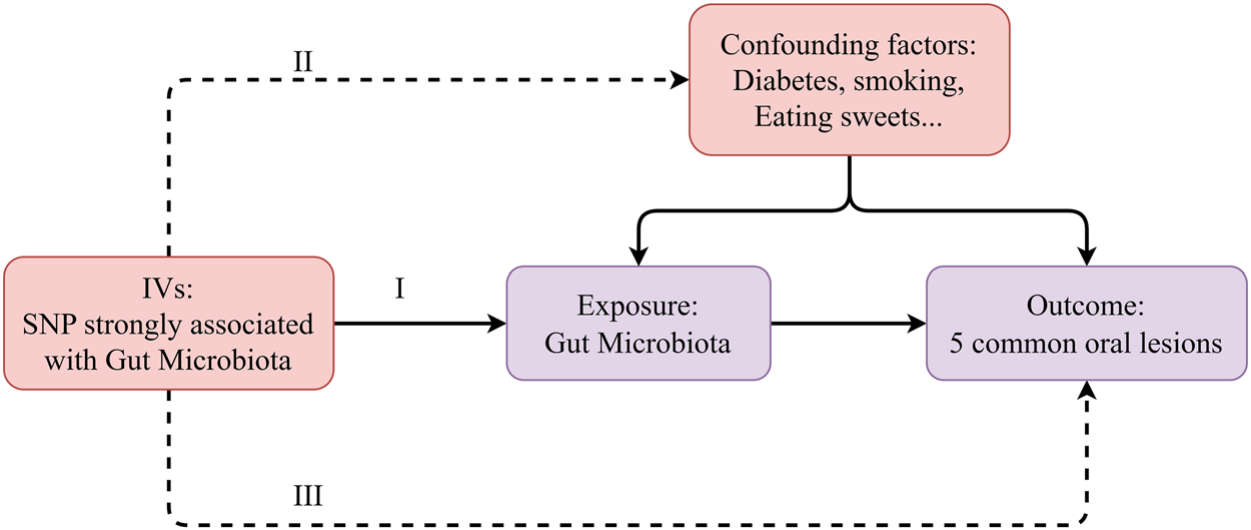
Three basic requirements for MR analysis. IVs = instrumental variables; SNPs = single nucleotide polymorphisms.

**Figure 2.**
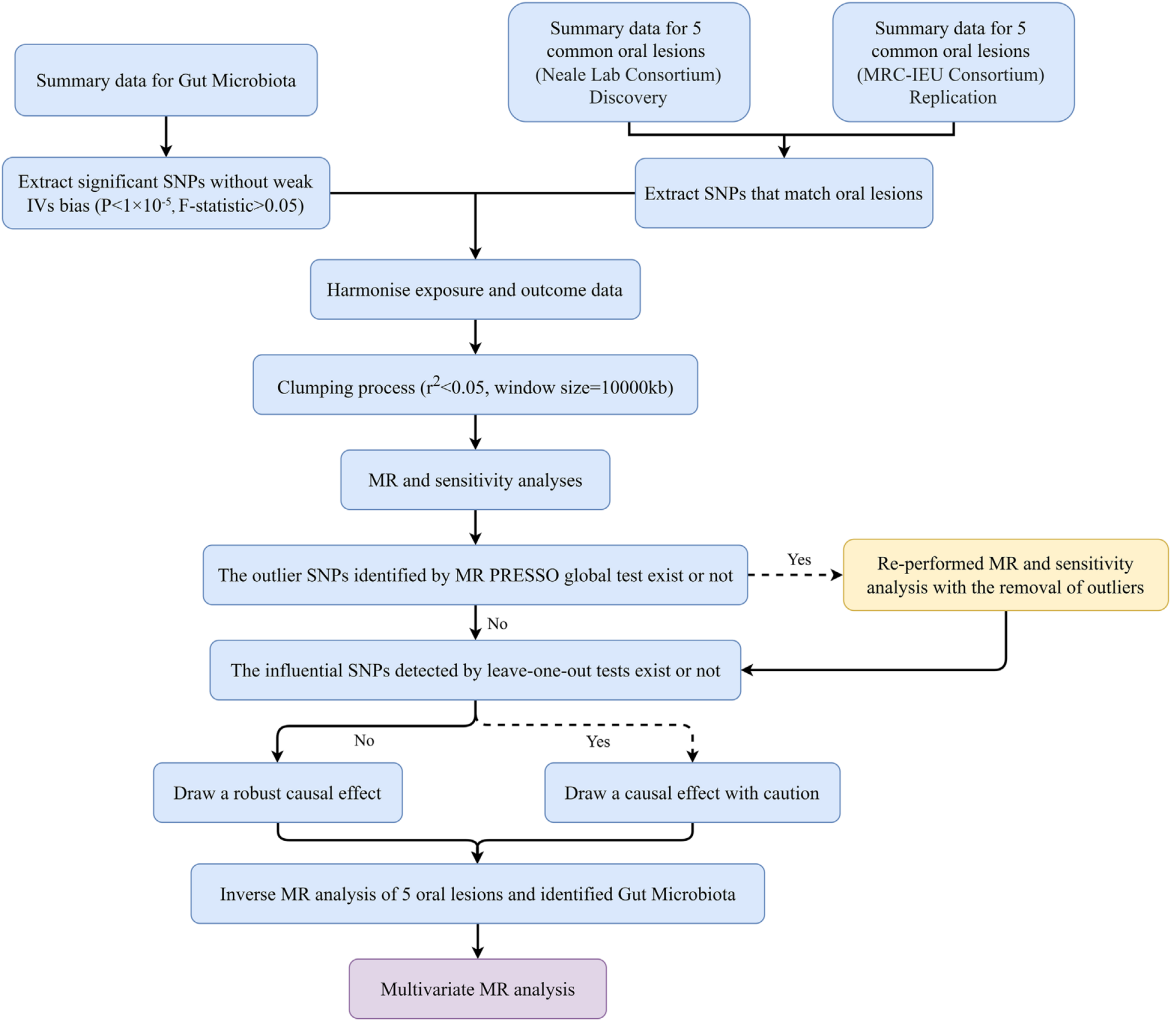
Flowchart outlining the MR analysis process. Two-sample MR was conducted to investigate the genetic links between gut microbiota and 5 oral lesions. Inverse MR analysis was then performed to assess the associations between oral lesions and the identified GM taxa. Finally, multivariate MR was applied to determine whether the effects of the identified GM taxa on the 5 oral lesions were direct or mediated through other factors.

Two-sample MR was utilized to investigate the genetic associations between GM taxa and 5 oral conditions. The summary data for this study were drawn from available genome-wide association studies (GWAS). The selection of SNPs as IVs followed stringent criteria, ensuring 3 essential conditions were met: the IVs must demonstrate a strong association with the exposure; the selected SNPs must be independent of the outcomes and any potential confounders; and the SNPs should influence the outcome solely through the exposure, without involvement in other biological pathways. These criteria are depicted in Figure 1. For GM taxa showing significant causal associations with oral lesions, we also conducted pre-specified bidirectional MR to evaluate the reverse causal effects of oral lesions on GM taxa. Subsequently, MVMR was performed to determine the independent causal effects of GM on oral lesions after accounting for traditional risk factors. The flowchart outlining the MR analysis process is illustrated in Figure 2. This study followed the STROBE-MR reporting guidelines.

### 2.2. Data sources for GMs

The summary statistics on GM composition were obtained from the GWAS Catalog (https://www.ebi.ac.uk/gwas/, ID: GCST90016908-GCST90017118) and were conducted by the MiBioGen consortium through a meta-analysis of 16S fecal microbiome data from 18,340 participants across 24 cohorts in Germany, Canada, the USA, Israel, Finland, South Korea, Belgium, Denmark, the Netherlands, Sweden, and the UK.^[27]^ The cohorts were of multi-ancestry but predominantly European origin. The data included a total of 211 GM taxa (131 genera, 35 families, 20 orders, 16 classes, and 9 phyla) with a mean abundance exceeding 1%. Fifteen bacterial taxa were excluded from the MR study due to a lack of available nomenclature, leaving 196 bacterial taxa (9 phyla, 16 classes, 20 orders, 32 families, and 119 genera) for inclusion in the analysis. We analyzed taxa across multiple levels of taxonomic resolution (from phylum to genus) to capture potential associations that may manifest differently at distinct microbial hierarchies. Detailed sample sizes and SNP numbers are provided in Table S1, Supplemental Digital Content 1, and further methodological details are available in previous reports.^[27]^

### 2.3. Data sources for oral lesions

GWAS summary data for 5 common oral lesions, loose teeth, toothache, bleeding gums, painful gums, and mouth ulcers, were sourced from the IEU OPEN GWAS project (https://gwas.mrcieu.ac.uk/), drawing on data from the Neale Lab and MRC-IEU consortium. The Neale Lab GWAS datasets were used as discovery datasets, based on self-reported phenotypes collected via UK Biobank questionnaires. Specifically, the datasets included loose teeth (GWAS ID: ukb-a-431, 13,238 cases and 322,900 controls), toothache (GWAS ID: ukb-a-432, 13,290 cases and 322,848 controls), bleeding gums (GWAS ID: ukb-a-430, 43,469 cases and 292,669 controls), painful gums (GWAS ID: ukb-a-429, 9287 cases and 326,851 controls), and mouth ulcers (GWAS ID: ukb-a-427, 34,398 cases and 301,740 controls). For validation, we used MRC-IEU GWAS datasets (n = 461113) comprising the same traits: loose teeth (ukb-b-12849), toothache (ukb-b-19191), bleeding gums (ukb-b-7872), painful gums (ukb-b-11161), and mouth ulcers (ukb-b-6458). Detailed information is provided in Table S2, Supplemental Digital Content 2.

### 2.4. Selection of IVs

Independent SNPs strongly associated with GM taxa (*P* < 5 × 10^−8^, r^2^ < 0.001, clumping window = 10,000 kb) were initially selected as candidate IVs. However, for many GM taxa the number of genome-wide significant SNPs was limited. Therefore, we adopted a relaxed threshold (*P* < 1 × 10^−5^) to ensure adequate instrument coverage and reduce weak instrument bias. To further avoid weak instruments bias, SNPs with F-statistics < 10 were excluded (F = β^2^/SE^2^). Potential confounder-related SNPs were identified and excluded using the LDtrait tool (https://ldlink.nih.gov/). Traditional risk factors for oral lesions, such as smoking, diabetes, and sugar intake, were treated as confounders.^[28,29]^ For reverse MR analyses, oral lesion-associated SNPs were selected using thresholds of *P* < 5 × 10^−8^ or *P* < 5 × 10^−6^ with the same clumping procedure. Additionally, all well-known risk factors influencing GM taxa, including aging, hormone intake, and antibiotic use, were treated as potential confounders and excluded from subsequent MR analysis. Exposure and outcome data were harmonized by aligning effect alleles, and palindromic SNPs with intermediate allele frequencies (MAF: 0.42–0.58) were removed to avoid strand ambiguity.

### 2.5. MR analysis

Two-sample MR was conducted to assess the causal relationships between GM and 6 common oral lesions. Five different methods were employed to obtain MR estimates: MR Egger regression, weighted median method, inverse variance weighted (IVW) method, simple mode method, and weighted mode method. The IVW method was used as the primary approach because it provides the most precise estimates under the assumption that all selected IVs are valid.^[30]^ MR Egger regression, while potentially less precise, is valuable for evaluating horizontal pleiotropy through Egger intercept values, even when all SNPs are invalid.^[31]^ The weighted median method was also utilized, as it can yield consistent estimates even if up to 50% of the selected SNPs are invalid IVs.^[32]^ Additionally, the simple mode and weighted mode methods served as supplementary techniques to further validate the causal estimates. To address multiple testing, adjusted P values for various GM taxa were applied using Bonferroni correction (0.05/n, where n is the number of GM taxa at each taxonomic level). The significance thresholds for the 5 taxa levels were established as follows: genera *P* = 3.82 × 10^-4^ (0.05/131), families *P* = .0014 (0.05/35), orders *P* = .0025 (0.05/20), classes *P* = .0031 (0.05/16), and phyla *P* = .0056 (0.05/9). For the potential associations between GM and oral lesions, inverse MR analysis was performed to investigate the causal effects of oral lesions on the identified GM composition.

To ensure the robustness and reliability of the identified associations, a series of sensitivity tests were conducted. First, Cochran Q test was used to evaluate potential heterogeneity in the associations. Next, we employed the MR-Egger Intercept and MR Pleiotropy Residual Sum and Outlier (MR-PRESSO) global tests to detect any horizontal pleiotropy. Additionally, MR-PRESSO Outlier and Distortion tests were utilized to identify and correct for horizontal pleiotropy by excluding outlier SNPs from the analysis. If outliers were detected, the MR analyses were re-run without these outliers to assess the stability of the results. Finally, a leave-one-out analysis was performed to determine whether the exclusion of individual SNPs would impact the significant associations.

The abundance of distinct GM taxa is influenced by various lifestyle factors and physical conditions. A straightforward 2-sample MR analysis may not fully capture the direct impact of GM on oral lesions, as these external factors can also contribute to the development of oral lesions.^[33]^ To disentangle direct versus mediated effects, MVMR was conducted adjusting for smoking, diabetes, and alcohol consumption.^[34]^

All statistical analyses were 2-sided and conducted using R software (version 4.2.3) with the R packages “TwoSampleMR,” “ieugwasr,” and “MRPRESSO.”

## 3. Results

### 3.1. Selection of IVs and overview

**Figure 3.**
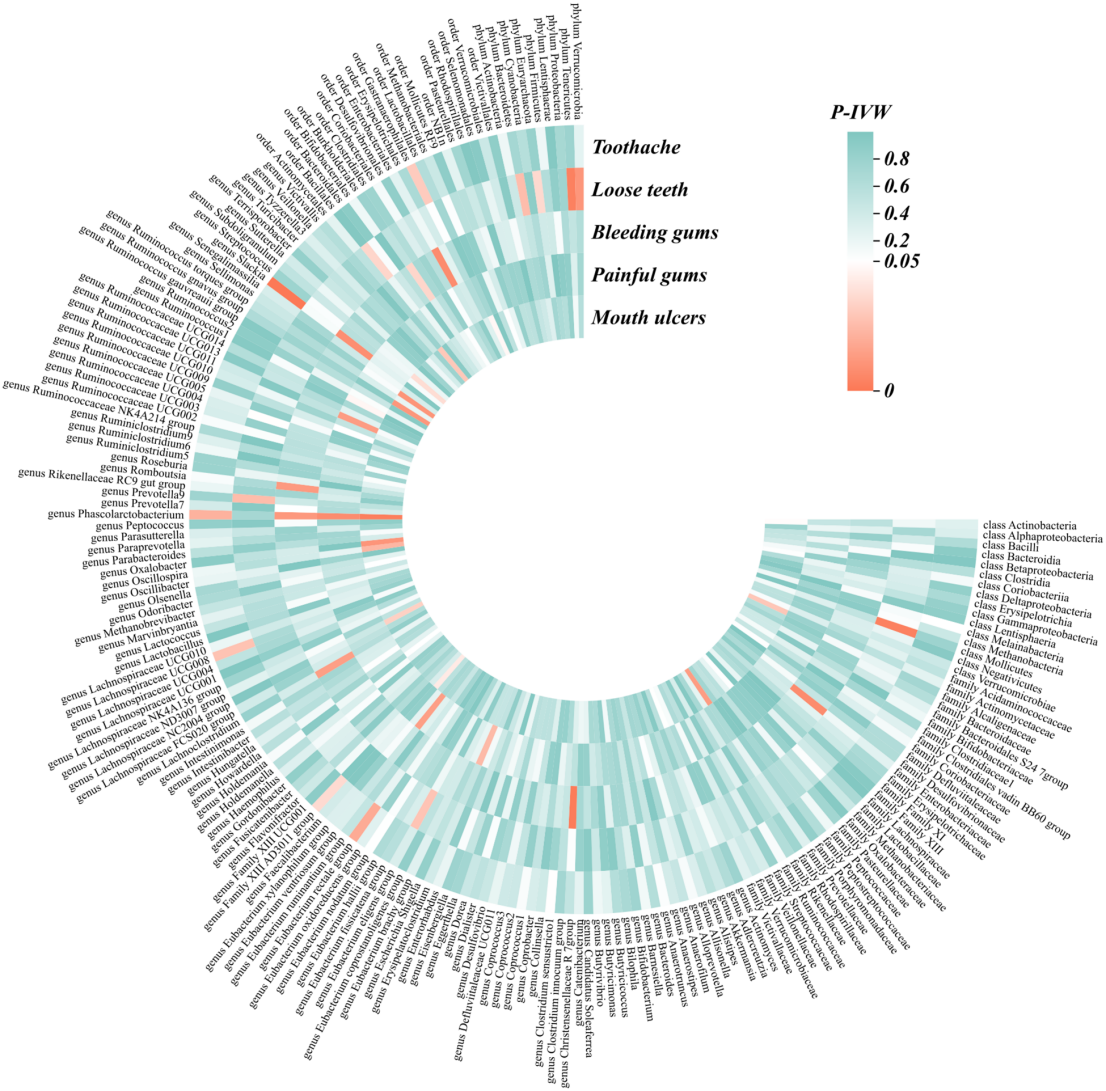
IVW analysis results showing the causal associations between gut microbiota and 5 oral lesions (Discovery Analysis). IVW = inverse variance weighted.

After selecting genome-wide independent SNPs (r^2^ < 0.001, clumping window size = 10,000 kb) and significant SNPs (*P* < 1 × 10^−5^), the number of IVs for the 196 GM taxa ranged from 1 to 25. In the reverse MR analysis, the number of IVs for the 5 oral lesions at the discovery datasets ranged from 10 to 35. Detailed information on each SNP can be found in Table S3, Supplemental Digi. Additionally, the lowest F-statistic among these SNPs was 16.9128, indicating a low likelihood of weak instrument bias. Subsequently, SNPs associated with the outcome and confounders were excluded using the LDtrait Tool. Palindromic SNPs with intermediate allele frequencies were then removed by aligning the effect alleles of the exposure and outcome data. Following this rigorous selection process, the final number of selected IVs ranged from 3 to 77 for GM taxa and oral lesions. Detailed information on all the SNPs ultimately used for the 196 GM taxa and 5 oral lesions is provided in Tables S4 and S5, Supplemental Digital Content 4, respectively. The overall analysis results obtained using the IVW method are presented in Figure 3.

### 3.2. Two-sample MR results

**Figure 4.**
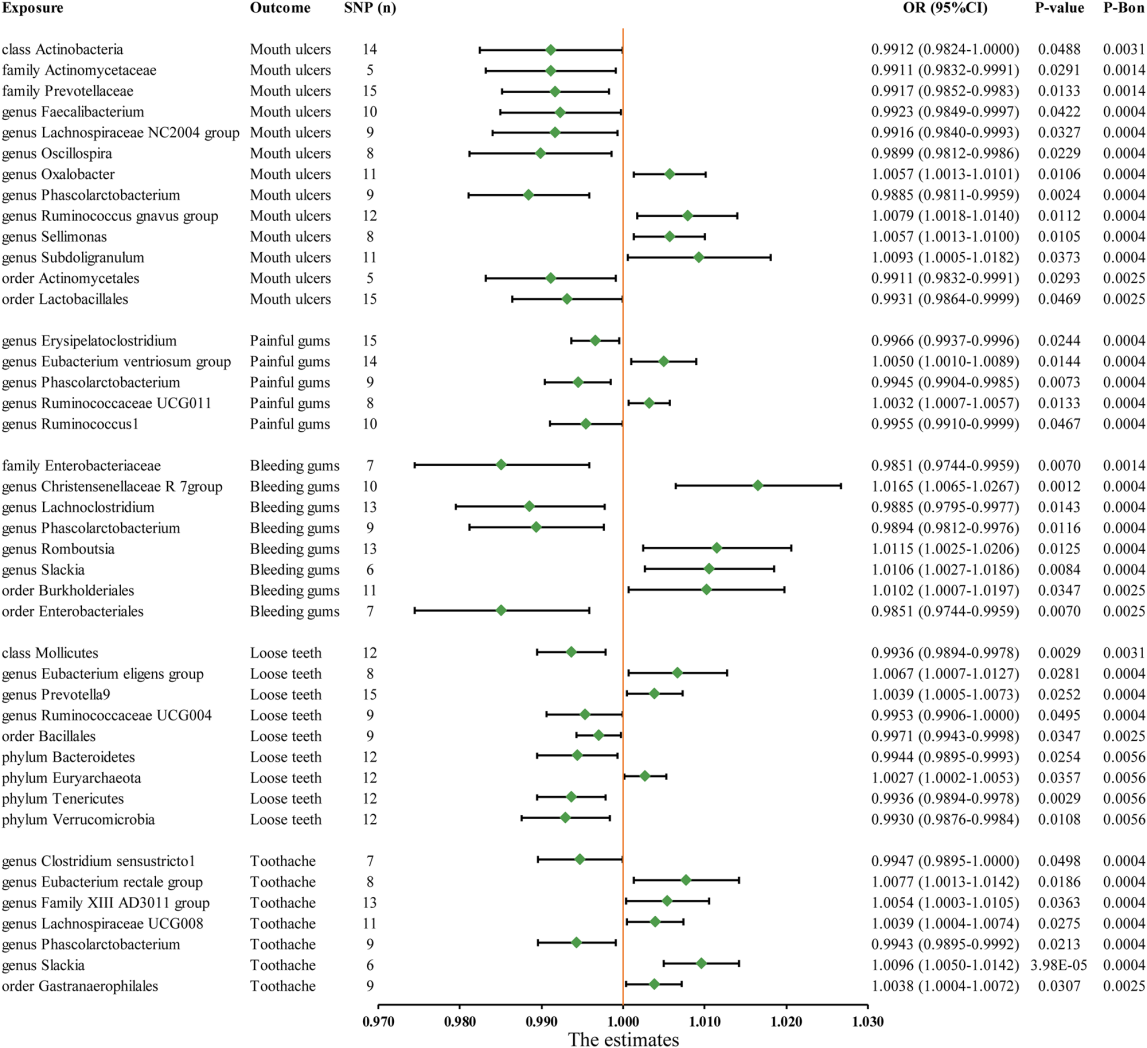
Initially significant IVW analysis results of gut microbiota on the 5 common oral lesions (Discovery Analysis). IVW: Inverse variance weighted; OR: Odds ratio; CI: Confidence interval; SNP (n): The number of single nucleotide polymorphisms; P-Bon: *P* value after Bonferroni correction, and results surviving multiple testing correction at *P* = .05/n, where n is the number of GM taxa at each taxonomic level.

**Figure 5.**
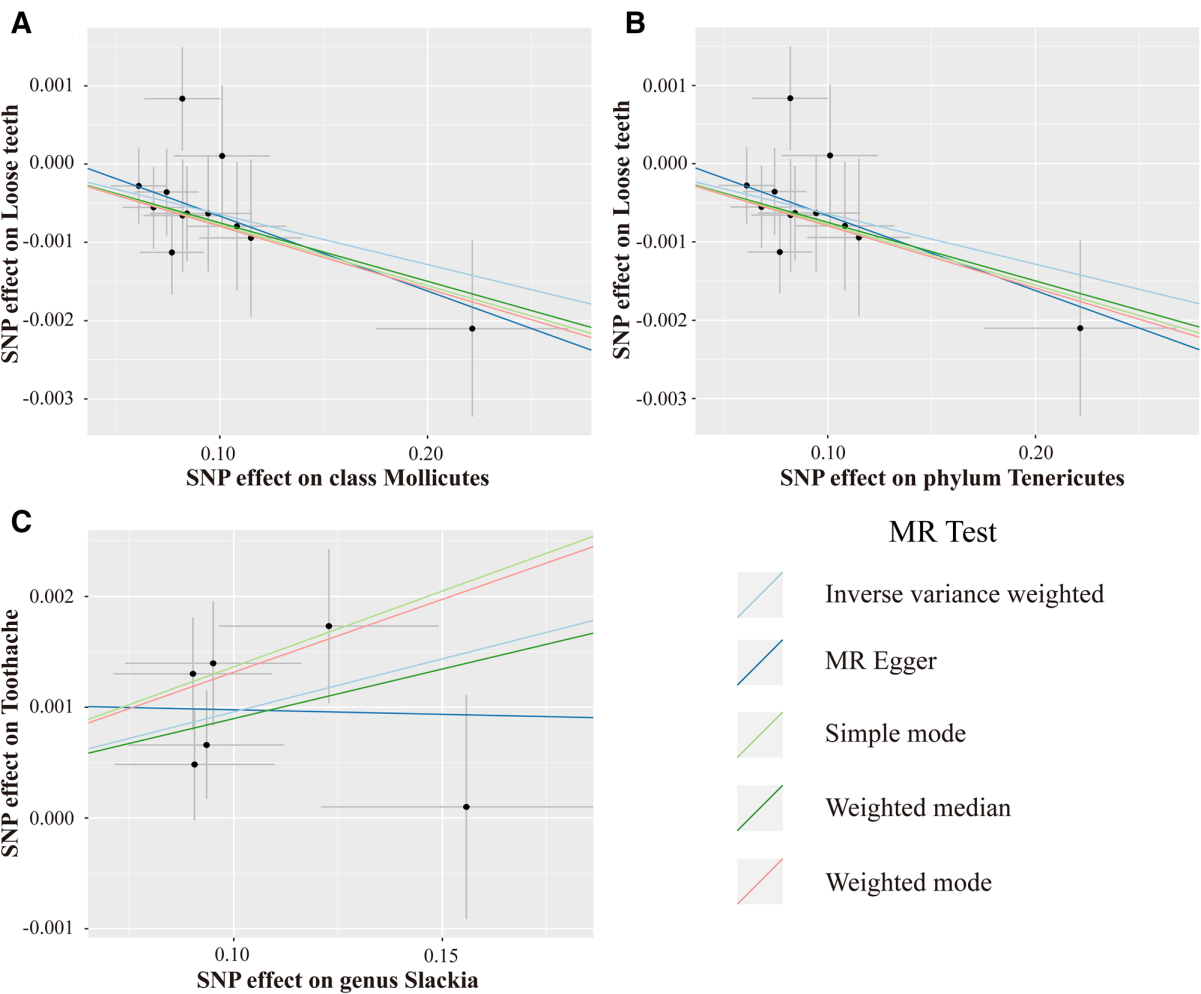
Scatter plots of significant associations between gut microbiota and oral lesions (Discovery Analysis). The line’s slope indicates the causal impact derived from each analysis method. (A) Mollicutes class on loose teeth; (B) Tenericutes phylum on loose teeth; (C) Slackia genus on toothache.

Using the IVW method, 42 associations reached nominal significance (*P* < .05) across the five oral lesions (Fig. 4). However, only 3 associations survived from Bonferroni correction. Specifically, the Mollicutes class and Tenericutes phylum were negatively associated with loose teeth (Mollicutes: OR = 0.9936, 95%CI: 0.9894–0.9978, *P* = .0029; Tenericutes: OR = 0.9938, 95%CI: 0.9892–0.9984, *P* = .0042), and the Slackia genus was positively associated with toothache (OR = 1.0096, 95%CI: 1.0050–1.0142, *P* = 3.98 × 10^-5^). Scatter plots of these associations are shown in Figure 5. Although OR values were close to 1, these results suggest subtle but biologically relevant effects, which may be meaningful at the population level in chronic conditions such as oral lesions.

**Figure 6.**
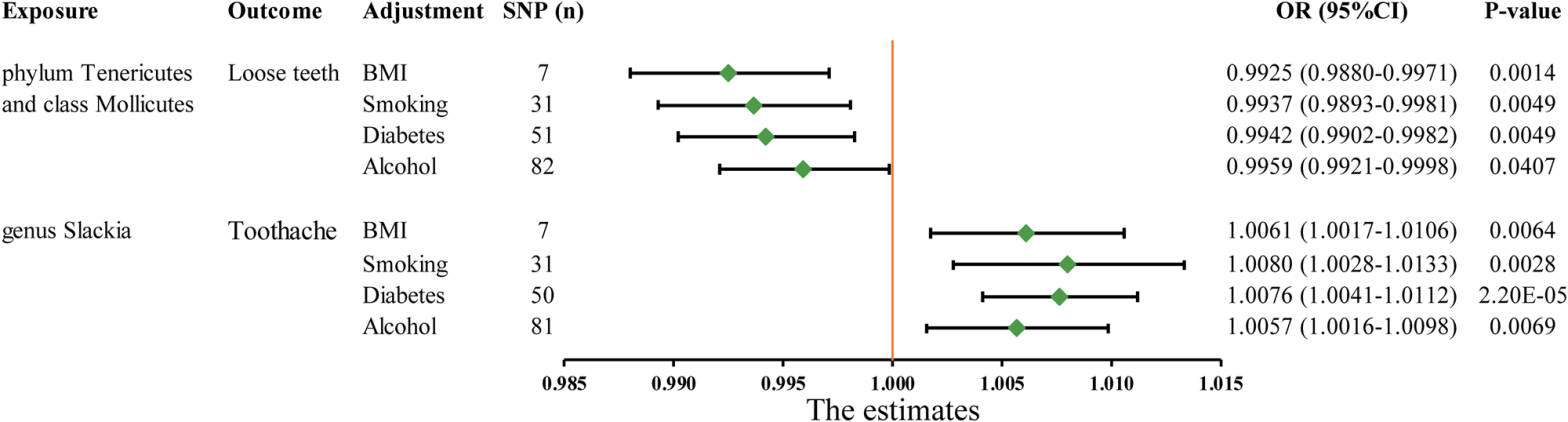
Replication results for 42 discovery-positive associations in the validation cohort. IVW = inverse variance weighted; OR = odds ratio; CI = confidence interval; SNP (n): The number of single nucleotide polymorphisms; P-Bon: *P* value after Bonferroni correction, and results surviving multiple testing correction at *P* = .05/n, where n is the number of gut microbiota taxa at each taxonomic level.

Additional complementary methods (including MR-Egger, weighted median, weighted mode, simple mode) supported the IVW findings or showed consistent trends (Table S6, Supplemental Digital Content 5). Reverse MR analyses indicated no significant causal effects of oral lesions on GM (Table S7, Supplemental Digital Content 6). In replication using MRC-IEU data, the 3 significant associations remained statistically significant and directionally consistent (*P* < .05), while 20 of the 42 initially suggestive associations were supported (Table S8, Supplemental Digital Content 7). A visual summary of association direction and strength is presented in the Figure 6.

For the 3 Bonferroni-significant associations, Cochran Q, MR-Egger intercept and MR-PRESSO tests showed no evidence of heterogeneity and horizontal pleiotropy (All *P* > .05; no outliers detected) in Table 1. The overall results of the sensitivity analysis are summarized in Table S9, Supplemental Digital Content 8. Leave-one-out analyses confirmed robustness (Figure S1, Supplemental Digital Content 9), and funnel plots indicated no directional bias (Figure S2, Supplemental Digital Content 10).

**Table 1:** Sensitivity analysis results for 3 significant associations after Bonferroni correction.

Exposure	Outcome	Cochran Q test		Egger Intercept test		MR-PRESSO	
		Q-IVW	*P*-value	Egger-intercept	*P*-value	*P*-value of Global test	*P*-value of distortion test
class Mollicutes	Loose teeth	6.9422	.8037	0.0003	.6416	.813	NA
phylum Tenericutes	Loose teeth	6.9422	.8037	0.0003	.6416	.835	NA
genus Slackia	Toothache	4.8453	.4350	0.0011	.5087	.434	NA

IVW = inverse variance weighted, MR = Mendelian randomization, MR-PRESSO = MR Pleiotropy Residual Sum and Outlier.

Regarding biological specificity, Slackia was associated with toothache but not bleeding gums or mucosal lesions. We speculate that Slackia may influence pain-related inflammatory pathways rather than bleeding or mucosal barrier integrity, explaining the observed pattern.

### 3.3. Multivariate MR results

**Figure 7.**
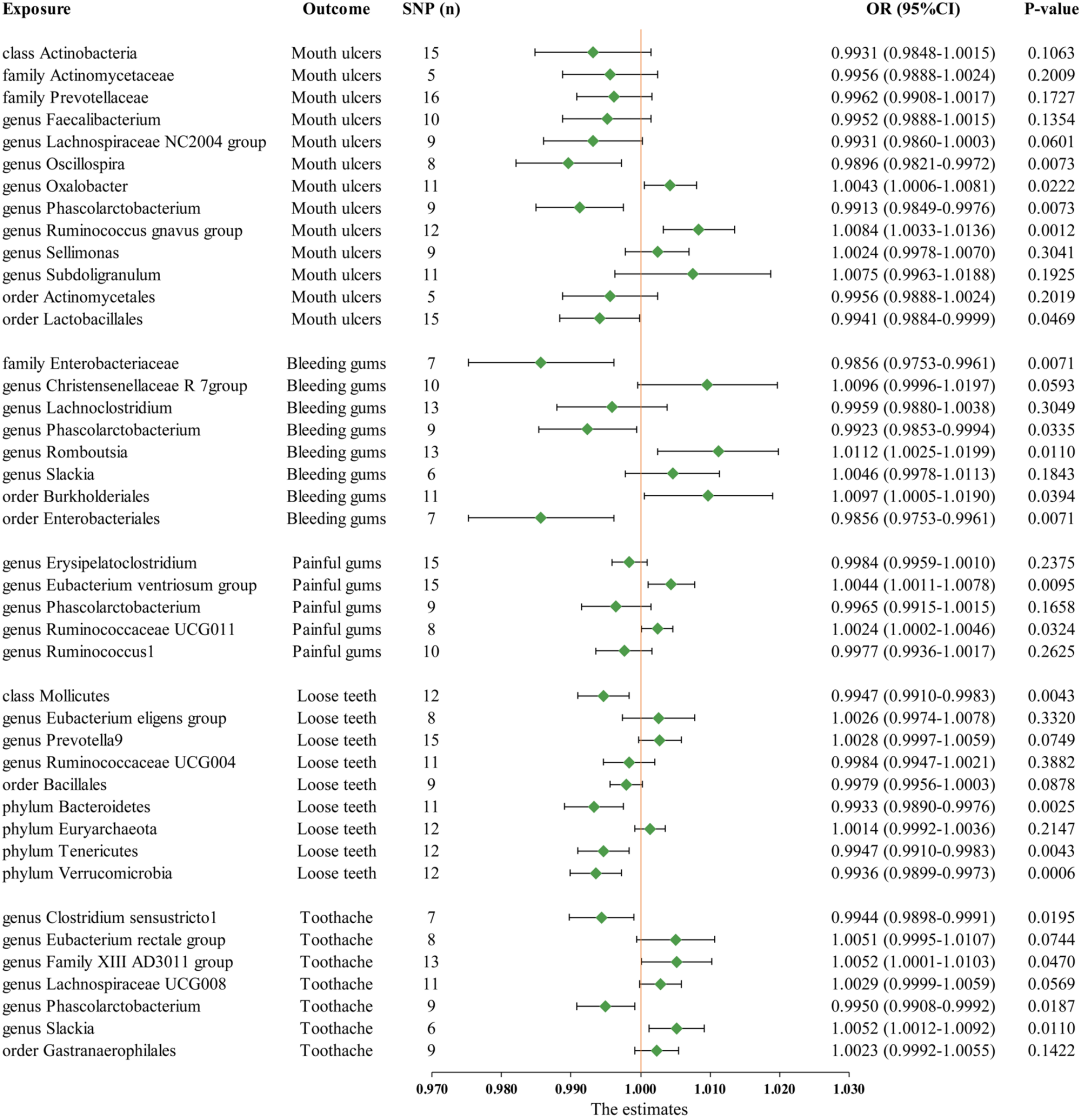
Multivariate MR results post-adjustment for confounding factors such as alcoholic drink consumption, diabetes, body mass index, and smoking. BMI = body mass index; OR = odds ratio; CI = confidence interval; SNP (n): The number of single nucleotide polymorphisms.

The objective of the MVMR analysis was to determine whether the significant impact of GM on oral lesions was direct or influenced by shared lifestyle factors or physical conditions, such as tobacco use, alcohol consumption, and diabetes. After adjusting for these potential confounders, the Mollicutes class and Tenericutes phylum maintained a negative association with the risk of loose teeth, while the Slackia genus remained positively associated with toothache, as shown in Figure 7. These results indicate that the observed effects are unlikely to be fully explained by common lifestyle risk factors.

## 4. Discussion

This study systematically investigated the causal associations between GM and 5 common oral lesions–loose teeth, toothache, bleeding gums, painful gums, and mouth ulcers–using MR. Our analysis genetically identified 42 GM taxa nominally associated with oral lesions, including 27 at the genus level, 3 at the family level, 6 at the order level, 2 at the class level, and 4 at the phylum level, with 3 associations surviving multiple testing correction: the Mollicutes class and Tenericutes phylum as protective factors for loose teeth, and the Slackia genus as a risk factor for toothache. Importantly, reverse MR analysis revealed no evidence of reverse causality from oral lesions to GM, underscoring the directionality of these associations.

Our findings align with and extend previous observational and GM sequencing studies. For example, reduced abundance of Mollicutes and Tenericutes has been observed in inflamed periodontal sites,^[35,36]^ consistent with their protective role suggested here. Conversely, Slackia has been implicated in peri-implant infections and dental caries in case–control studies,^[37,38]^ in line with our observation of its association with toothache. However, our MR design minimizes confounding and reverse causation, providing stronger inferential evidence compared with traditional observational designs.

The biological plausibility of these associations deserves attention. Loose teeth are prevalent among the elderly, individuals with diabetes, smokers, those with lower educational levels, and people with poor oral hygiene. The pathogenesis of loose teeth is complex, involving microbial dysbiosis, host immune responses, oxidative stress, and inflammation.^[39]^ Mollicutes and Tenericutes, characterized by wall-less bacterial structures and limited biosynthetic capacity, may confer protective effects by producing metabolites such as acetic acid and hydrogen, which have anti-inflammatory properties.^[40–42]^ Toothache, defined as pain originating from a tooth and its supporting structures, is the most common form of orofacial pain.^[43,44]^ Its pathogenesis is complex, involving the interplay of factors such as microbial imbalance, host immune response, oxidative stress, and inflammation, which collectively lead to the destruction of periodontal tissues and subsequent pain.^[45,46]^ Slackia, a gram-positive anaerobe, has been reported in bacteremia, intestinal abscess, and peri-implant infections.^[47,48]^ Its metabolic features and potential virulence factors may disrupt the oral microbiome and exacerbate inflammatory responses, thereby contributing to dental pain. While these mechanisms remain speculative, they provide testable hypotheses for future experimental studies. Beyond toothache, the potential role of Slackia in prosthodontic complications warrants consideration. Its association with peri-implantitis suggests possible implications for implant failure and denture stomatitis.^[37,38,49]^ Although our data cannot directly address these outcomes, the findings raise clinically relevant questions that could guide future research in dental and prosthodontic settings.

Several limitations must be acknowledged. First, although sensitivity analyses (Egger intercept, MR-PRESSO, MVMR) reduced the likelihood of pleiotropy, horizontal pleiotropy remains an inherent limitation in MR studies. Second, weak instrument bias cannot be completely excluded, despite all F-statistics exceeding the conventional threshold. Third, our GWAS datasets included only individuals of European ancestry, limiting the generalizability of our findings to other populations. Another limitation is that the statistical power may be insufficient to detect modest causal effects, particularly given the relatively low effect sizes observed in our analysis. Moreover, the observed effect sizes (ORs close to 1.0) suggest that while statistically robust, the clinical magnitude of these associations may be modest, and they should be interpreted with caution. Nonetheless, this study has notable strengths. We leveraged large GWAS consortia, used multiple complementary MR methods, and replicated findings in an independent dataset. The null results from reverse MR further reduce the risk of reverse causality, supporting the credibility of the identified associations. Future studies should pursue prospective oral microbiome cohorts, mechanistic research into microbial metabolites and host interactions, and randomized controlled trials targeting gut–oral microbial pathways through interventions such as prebiotics or probiotics. Expanding genetic studies to non-European populations will also be critical to assess global relevance.

## 5. Conclusions

In conclusion, our study has suggested potential causal associations between specific bacterial taxa and common oral lesions using MR analysis. Notably, the Mollicutes class and Tenericutes phylum were found to be negatively associated with loose teeth, suggesting a protective role against this condition. Additionally, the Slackia genus showed a positive association with toothache, indicating its potential involvement in the etiology of this condition. These findings offer preliminary insights into the potential role of the GM in oral health, while highlighting areas that warrant further exploration. However, further research is needed to validate the specific contributions of GM taxa to the pathogenesis of oral lesions and to deepen our understan ding of the underlying disease mechanisms.

## Acknowledgments

All genetic data were sourced from the GWAS catalog and IEU Open GWAS project. We extend our gratitude to all participants and coordinators who contributed to the data used in this study.

## Author contributions

**Conceptualization:** Ying Tang, Jianming Yuan, Feng Pan.

**Data curation:** Ying Tang, Jianming Yuan.

**Formal analysis:** Ying Tang, Jianming Yuan.

**Investigation:** Ying Tang, Jianming Yuan, Feng Pan.

**Methodology:** Ying Tang, Jianming Yuan.

**Project administration:** Feng Pan.

**Resources:** Ying Tang, Jianming Yuan.

**Software:** Ying Tang, Jianming Yuan.

**Supervision:** Feng Pan.

**Validation:** Jianming Yuan, Feng Pan.

**Visualization:** Ying Tang, Jianming Yuan.

**Writing – original draft:** Ying Tang.

**Writing – review & editing:** Ying Tang, Feng Pan.






















